# Combined IFN-γ and IL-2 release assay for detect active pulmonary tuberculosis: a prospective multicentre diagnostic study in China

**DOI:** 10.1186/s12967-021-02970-8

**Published:** 2021-07-03

**Authors:** Yaoju Tan, Yunhong Tan, Junlian Li, Pengnan Hu, Ping Guan, Haobin Kuang, Qide Liang, Yanyan Yu, Zhongnan Chen, Quan Wang, Zhenping Yang, DiLiNaZi AiKeReMu, Yu Pang, Jianxiong Liu

**Affiliations:** 1 Clinical Laboratory, Guangzhou Chest Hospital, Guangzhou/State Key Laboratory of Respiratory Diseases, Guangzhou, China; 2 Clinical Laboratory, Hunan Chest Hospital, Changsha, China; 3 Clinical Laboratory, Chest Hospital of Xinjiang Uygur Autonomous Region, Ürümqi, China; 4 School of Life Science & Technology, LingNan Normal University, Zhanjiang, China; 5 Department of Bacteriology and Immunology, Beijing Key Laboratory on Drug-Resistant Tuberculosis Research, Beijing Chest Hospital, Capital Medical University/Beijing Tuberculosis & Thoracic Tumor Research Institute, Beijing, China

**Keywords:** Diagnosis, Interferon-γ, Interleukin-2, Tuberculosis

## Abstract

**Background:**

We performed a prospective multicentre diagnostic study to evaluate the combined interferon-γ (IFN-γ) and interleukin-2 (IL-2) release assay for detect active pulmonary tuberculosis (TB) in China.

**Methods:**

Adult patients presenting symptoms suggestive of pulmonary TB were consecutively enrolled in three TB-specialized hospitals. Sputum specimens and blood sample and were collected from each participant at enrolment. The levels of *Mycobacterium tuberculosis* (MTB)-specific antigen-stimulated IFN-γ and IL-2 were determined using enzyme-linked immunosorbent assay (ELISA).

**Results:**

Between July 2017 and December 2018, a total of 3245 patients with symptoms suggestive of pulmonary TB were included in final analysis. Of 3245 patients, 2536 were diagnosed as active TB, consisting of 1092 definite TB and 1444 clinically diagnosed TB. The overall sensitivity and specificity of IFN-γ were 83.8% and 81.5%, respectively. In addition, compared with IFN-γ, the specificity of IL-2 increased to 94.3%, while the sensitivity decreased to 72.6%. In addition, the highest sensitivity was achieved with parallel combination of IFN-γ/IL-2, with a sensitivity of 87.9%, and its overall specificity was 79.8%. The sensitivity of series combination test was 68.5%. Notably, the sensitivity of series combination test in definite TB (72.1%) was significantly higher than that in clinically diagnosed TB (65.8%).

**Conclusion:**

In conclusion, we develop a new immunological method that can differentiate between active TB and other pulmonary diseases. Our data demonstrates that the various IFN-γ/IL-2 combinations provides promising alternatives for diagnosing active TB cases in different settings. Additionally, the diagnostic accuracy of series combination correlates with severity of disease in our cohort.

## Background

Tuberculosis (TB), caused by infection with the bacterium *Mycobacterium tuberculosis* (MTB) complex, remains a major cause of mortality and morbidity worldwide, especially in developing countries [1, 2]. According to recent estimates, there were 10.0 million incident TB cases and 1.4 million TB deaths in 2018, raising a global public health concern [2]. Early and accurate diagnosis of TB is essential to initiate timely and proper treatment, and reduce TB transmission in the community [3]. However, of the estimated 10.0 million new TB cases, only 7.2 million were diagnosed [2]. The diagnostic gap is majorly caused by the lack of highly sensitive and accessible methods [4]. Recently, WHO recommended use of molecular diagnostics as the initial test for tuberculosis to increase case detection, such as GeneXpert MTB/RIF and TB-LAMP [5, 6]. Despite their promising efficacy in detecting culture-positive TB patients, they have insufficient sensitivity to provide confirmed evidence for culture-negative patients that account for half of TB burden [4]. In view of the paucibacillary nature of most culture-negative cases, this rigorous challenge highlights the urgent need to develop novel immunological diagnostics based on blood test rather than direct detection of the bacteria or nucleic acids [4].

Over the past decade, advances in host immune mechanisms against tubercle bacilli have facilitated the development of new immunological tests [7]. Of these, interferon-γ (IFN-γ) release assays (IGRAs), immune-based blood tests that measure T-cell responses to MTB-specific antigens, are widely used for detecting MTB infection [8]. The high accuracy of these assays relies upon detection of IFN-γ, which is considered as the most important cytokine secreted by type 1 (Th-1) T cell response in host to effectively control the infection with MTB [9]. Although IGRAs have been established as the gold standard for diagnosing latent tuberculosis infection (LTBI), they are not endorsed for differentiating active TB from LTBI [10]. Recently, besides ESAT-6 and CFP-10, the new generation of IGRA includes the new MTB-specific secreted protein TB 7.7 as antigen that boosts the host cellular immune response, thereby achieving the increased sensitivity for identifying LTBI [11]. However, the unvarying detection principle hinders its application in diagnosing of active cases or predicting the risk of developing active disease. Discovery of other immunological biomarkers thus presents an opportunity to develop the novel diagnostics to discriminate between active TB and LTBI [3].

Besides IFN-γ, interleukin-2 (IL-2) is additional cytokine produced by Th1 cells, which stimulates both Th1 cells and cytotoxic T lymphocytes [9]. Evidence from previous studies suggests that the IL-2 plays important roles in protective immune responses against MTB infection, and increasing concentrations of IL-2 were observed in the body fluids of active TB individuals compared with control individuals [12]. However, discordant results concerning the role of IL-2 in predicting the active TB development from LTBI have been reported by various researchers [13, 14]. It’s worth noting that the previous conclusions were drawn based on small samples, which could weaken their reliability. Therefore, more clinical data is urgently required to elucidate the role of IL-2 to differentiate active TB from LTBI and other respiratory diseases. To address this concern, we performed a prospective multicentre diagnostic study to evaluate the combined IFN-γ and IL-2 release assay for detect active pulmonary tuberculosis in China.

## Materials and methods

### Participants

We conducted a prospective multicenter study in three TB-specialized hospitals in China, including Guangzhou Chest Hospital, Hunan Chest Hospital, and Xinjiang Uyghur Chest Hospital. Adult patients presenting symptoms suggestive of pulmonary TB were consecutively enrolled in the pilots, whereas those aged < 18 years and unwilling to provide informed consent were excluded from our study. Participants were first interviewed by clinical physicians at enrolment. Then a baseline blood sample was drawn and demographic data were collected by completion of a case report form. Participants were followed up for 6 months thereafter, in order to monitor the response to anti-TB treatment if initiated. For patients affected by non-TB disease, the final diagnosis was made by hospital clinicians according to medical record. The study was approved by the Ethical Committee of the Guangzhou Chest Hospital, and all participants gave their written informed consent.

### Definitions

Diagnoses of all participants were categorized into four groups on the basis of physical examination, laboratory results and the response to medications: (i) definite tuberculosis: microbiological culture or positive molecular test of MTB, clinical symptoms and radiological findings suggestive of TB; (ii) clinically diagnosed tuberculosis: clinical symptoms and radiological findings suggestive of TB plus appropriate response to anti-TB therapy; (iii) latent tuberculosis infection: positive IGRA results and no clinical evidence of active TB cases; (iv) Others: negative IGRA results and final diagnosis of other respiratory diseases. Active TB cases included definite and clinically diagnosed TB cases, while non-TB cases included LTBI and other diseases.

### Laboratory procedures

Sputum specimens and blood sample (8 mL) and were collected from each participant at enrolment. Sputum was stained with auramine O and examined for acid-fast bacilli using fluorescent microscopy according to guidelines of the National Tuberculosis Programme in China [15]. Specimens were processed immediately with the NALC method. After neutralization with PBS buffer, each suspension was centrifuged for 15 min at 3000×*g*. The resuspension of pellet was inoculated into a BACTEC MGIT tube (BD Microbiology Systems, USA) [16]. All positive cultures underwent species identification for MTB with the MPT64 antigen method, and the positive cultures without MPT64 expression were further identified by molecular method as previously reported [16]. Additionally, 1.0 mL of sputum specimen was digested with 2.0 mL of Xpert sample reagent. After incubation at room temperature for 15 min, 2.0 mL of inactivated mixture was pipetted into a Xpert MTB/RIF cartridge.

For blood sample, 3 mL was detected with QuantiFERON-TB Gold according to the manufacturer’s instructions. In addition, the peripheral blood mononuclear cells (PBMCs) of the remaining 4 mL blood sample were separated using lymphocyte cell separation media (TBD, Tianjin, China) within 4 h of blood withdrawal. Then the PBMCs with a density of 2.5 × 10^6^ cells/mL were stimulated with an ESAT-6–CFP-10-Rv1985c fusion protein (T), positive control phytohemagglutinin (PHA) (P), and negative control AIM-V medium (N) at 37 °C for 16–20 h. Cell culture supernatants were collected after 16–20 h of incubation for cytokine determination. The supernatant was stored at 20 °C until assays were performed. The levels of IFN-γ and IL-2 were determined using enzyme-linked immunosorbent assay (ELISA). The values of MTB-specific antigen-stimulated cytokines were calculated from TB antigen (T) minus negative control (N).

### Statistical analysis

The original data were entered into a computer by a double data entry method using the EpiData Entry data entry program (http://www.epidata.dk/). The input database was exposed to SPSS 20.0 for analysis. The Chi-square analysis was performed to investigate the distribution of TB cases stratified to different demographic characteristics across different groups. Student's t-test were conducted for continuous demographic variables. In addition, the cut-off values were determined by the receiver operating characteristic (ROC) curve analysis. *P* values less than 0.05 were interpreted as statistically significant. All statistical calculations were conducted using SPSS version 15.0 (SPSS, Chicago, IL).

## Results

### Participants

**Fig. 1:**
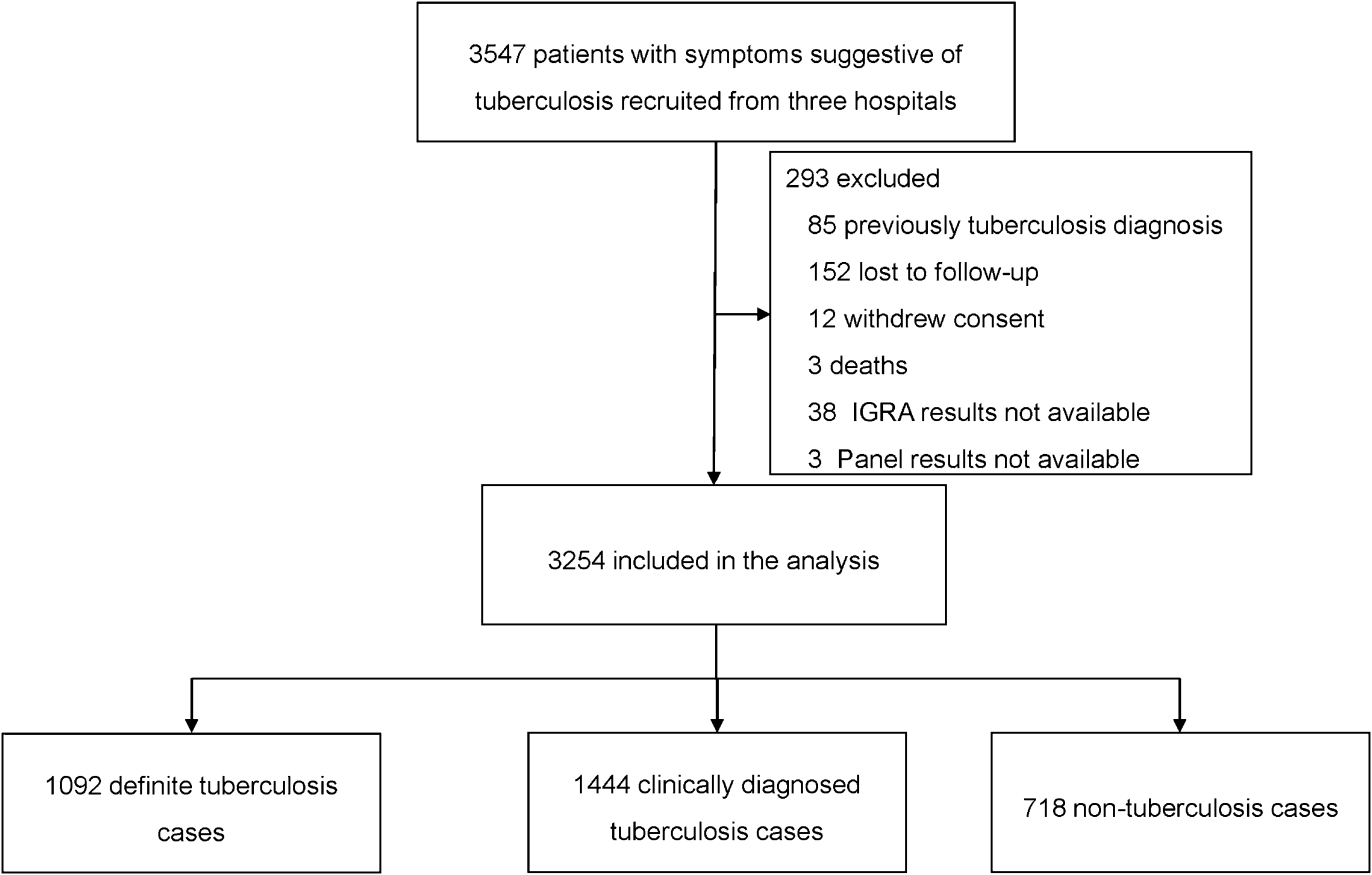
Enrolment of patients with symptoms suggestive of pulmonary tuberculosis

Between July 2017 and December 2018, a total of 3547 patients with symptoms suggestive of pulmonary TB were enrolled in the study. Of them, 293 (8.3%) were excluded from analysis on the basis of exclusion criteria, including 85 with a history of TB diagnosis, 152 lost to follow-up, 12 withdrew consent, 3 deaths, and 48 without available laboratory results. Finally, 3245 patients were included in final analysis (Fig. 1). Demographic and clinical characteristics of the study population are summarized in Table 1. The median age of the cohort was 52.0 years (IQR 33.0–65.0), and 61.1% of the patients were male. Of 3245 patients, 2536 were diagnosed as active TB, consisting of 1092 definite TB and 1444 clinically diagnosed TB. The reaming 718 non-TB cases were subclassified according to their final diagnosis. The pneumonia and lung cancer were the most frequently observed diseases, accounting 84.8% of non-TB cases. In addition, 223 (31.1%) out of 718 cases were classified as LTBI.

**Table 1:** Demographic and clinical characteristics of participants enrolled in this study

Characteristic^a^	No. of participants (%) (*N* = 3254)
Median age (IQR)—year	52.0 (33.0–65.0)
Male sex—no. (%)	1989 (61.1%)
Region	
Guangzhou	901 (27.7)
Hunan	1044 (32.1)
Xinjiang	1309 (40.2)
Classification	
Active TB	2536 (77.9)
Definite TB	1055 (33.6)
Clinically diagnosed TB	1481 (44.4)
Non-TB	718 (22.1)
Pneumonia	382 (11.7)
Lung cancer	227 (7.0)
Bronchiectasis	82 (2.5)
NTM	27 (0.8)

^a^*IQR* inter quartile range, *TB* tuberculosis, *NTM* nontuberculous mycobacteria

### Diagnostic utility of IFN-γ and IL-2 release assay

**Fig. 2:**
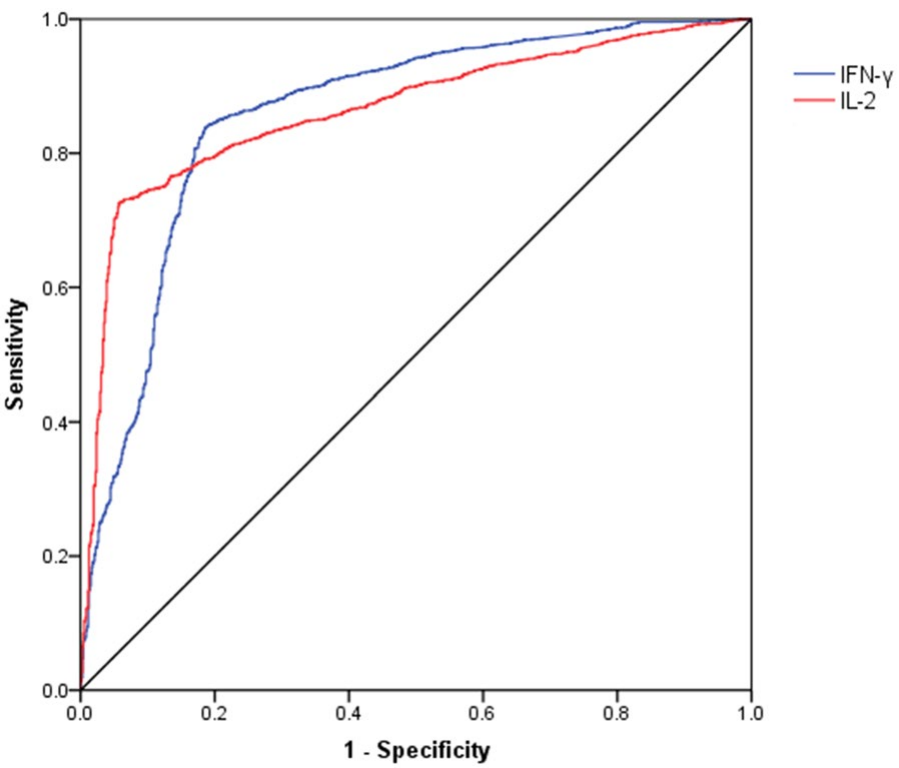
ROC curve of IFN-γ/IL-2 for differentiating active TB from non-TB

The ROC curves of IFN-γ and IL-2 release assay for diagnosing active TB cases were shown in Fig. 2. The area under ROC curve was 0.859 [95% confidence interval (95% CI) 0.842–0.875] for IFN-γ and 0.865 (95% CI 0.851–0.879) for IL-2. The optimal cut-off values for IFN-γ and IL-2 were determined as 7.38 ng/L and 20.19 ng/L, respectively. Based on these cutoff values, the corresponding sensitivity, specificity, positive predictive value and negative predictive value were summarized in Table 2. The overall sensitivity and specificity of IFN-γ were 83.8% (95% CI 82.2–85.2) and 81.5% (95% CI 78.4–84.2), respectively. In addition, compared with IFN-γ, the specificity of IL-2 increased to 94.3% (95% CI 92.3–95.8), while the sensitivity decreased to 72.6% (95% CI 70.8–74.3).

**Table 2:** Diagnostic accuracy of the MTB antigen-stimulated INF-γ and IL-2 for diagnosis of active tuberculosis

Cytokine	AUC (95% CI)	Cut-off value (pg/mL)	Sensitivity (%, 95% CI)	Specificity (%, 95% CI)	PPV (%, 95% CI)	NPV (%, 95% CI)
IFN-γ	0.859 (0.842–0.875)	7.38	83.8 (82.2–85.2)	81.5 (78.4–84.2)	94.1 (93.0–95.0)	58.7 (55.5–61.7)
IL-2	0.865 (0.851–0.879)	20.19	72.6 (70.8–74.3)	94.3 (92.3–95.8)	97.8 (97.0–98.4)	49.3 (46.6–52.0)

*AUC* area under curve, *CI* confidence interval, *PPV* positive predictive value, *NPV* negative predictive value

### Diagnostic utility of combined use of IFN-γ and IL-2

We further analyzed the diagnostic utility of combined use of IFN-γ and IL-2 for diagnosis of active TB cases. As shown in Table 3, the highest sensitivity was achieved with parallel combination that included IFN-γ and IL-2, with a sensitivity of 87.9% (95% CI 86.5–89.1), and its overall specificity was 79.8% (95% CI 76.6–82.6). When the series combination of IFN-γ and IL-2, 689 out of 718 non-TB were correctly identified, giving as specificity of 96.0% (95% CI 94.2–97.2). In addition, the overall sensitivity of series combination test was 68.5% (95% CI 66.6–70.3). Notably, the sensitivity of series combination test in definite TB (72.1%, 95% CI 69.3–74.8) was significantly higher than that in clinically diagnosed TB (65.8%, 95% CI 63.3–68.2; *P* = 0.001). Of the 1,055 definite TB patients, 579 (54.9%) had smear-negative results. Sensitivity of various combinations in this population were: 73.1% (95% CI 68.8–77.0) for series combination, and 90.1% (95% CI 87.0–92.6) for parallel combination.

**Table 3:** Diagnostic accuracy of the combined INF-γ and IL-2 for diagnosis of active tuberculosis cases

Classification	Series combination of INF-γ and IL-2		Parallel combination of INF-γ and IL-2	
	n/N	Estimate (95% CI)	n/N	Estimate (95% CI)
Sensitivity for active tuberculosis				
All active tuberculosis	1736/2536	68.5 (66.6–70.3)	2228/2536	87.9 (86.5–89.1)
Definite tuberculosis	761/1055	72.1 (69.3–74.8)	942/1055	89.3 (87.2–91.1)
Smear-positive tuberculosis	413/579	71.3 (67.4–74.9)	513/579	88.6 (85.7–91.0)
Smear-negative tuberculosis	348/476	73.1 (68.8–77.0)	429/476	90.1 (87.0–92.6)
Clinically diagnosed tuberculosis	975/1481	65.8 (63.3–68.2)	1286/1481	86.8 (85.0–88.5)
Specificity for active tuberculosis				
Active tuberculosis excluded	689/718	96.0 (94.2–97.2)	573/718	79.8 (76.6–82.6)
Active tuberculosis and LTBI excluded	495/495	100.0 (99.0–100.0)	470/495	94.9 (92.5–96.6)
Predictive values for all tuberculosis				
Positive predictive value	1736/1765	98.4 (97.6–98.9)	2228/2373	93.9 (92.8–94.8)
Negative predictive value	689/1489	46.3 (43.7–48.8)	573/881	65.0 (61.8–68.2)

## Discussion

Current diagnostic tests for tuberculosis remains challenging despite years of development [3]. The poor sensitivity of conventual culture and molecular tests highlights the requirement of novel diagnostics that providing an opportunity to diagnose active tuberculosis patients, especially those with paucibacterial specimens [17]. In this study, we develop a new immunological method that can differentiate between active TB and other pulmonary diseases. In a recent meta-analysis, Erascella et al. found that the asymptomatic TB accounted for a substantial proportion of disease burden [18]. These patents always had negative or paucibacillary sputum samples, highlighting the incapability to conventional methods to diagnose them. As an alternative, this TB-specific immunologic assay may provide more benefit for these asymptomatic individuals, thereby facilitating early diagnosis and timely initiation of appropriate therapy for pulmonary TB.

The sensitivity achieved 87.9% by the parallel use of TB-specific antigen stimulated the IFN-γ and IL-2, compared with 83.8% by the single use of IFN-γ. The increase in the sensitivity was majorly due to the introduction of IL-2 as the parallel diagnostic marker, indicating that approximate 16% of active TB cases have negative IFN-γ results, whereas one of quarter of these cases have positive IL-2 results. Similarly, in a recent systematic review, a pooled sensitivity of 81% was estimated for the active TB cases of the QFT-GIT [8]. Previous studies have proposed various aetiologies affecting the immunosuppression for false-negative IGRA test results, and therefore reduced reactivity to the assays [8]. In contrast, when setting the positive control (PBMCs stimulated with phytohemagglutinin) of our assays as indicators for the individual immune response, we could not identify the immunosuppression as risk factor for false-positive results (data not shown). A potential explanation for the negative IGRA results may be due to the compartmentalization of T cells [19]. Considering that the peripheral blood T cells are isolated for measuring IFN-γ levels in IGRA assays, the recruitment of the TB antigen-specific T cells at the sites of infections during the initial course of TB diseases may be associated with negative IGRA results.

Of note, despite failure to detect the excretion of IFN-γ, a small proportion of MTB-specific T cells only secreted IL-2 in our population. In line to our observation, an experimental study on dynamic relationship between IFN-γ and IL-2 profile during the nature of human tuberculosis demonstrated that newly detectable IL-2-only secreting CD4+ T cells during and after treatment [20], indicating its potential role for the differential diagnosis between active tuberculosis and LTBI. Our detailed analysis of MTB-specific cytokine excretion confirmed that IL-2 is more specific for active TB cases than IFN-γ. During the course of TB infection, the MTB-specific T cells express high level of PD-1 at the late phase of chronic infection, thus limiting their capacity to excrete IL-2 rather than IFN-γ [21], and this may be a plausible explanation for high specificity of IL-2 in TB diagnosis. When series combination of IFN-γ and IL-2, the specificity further increased to 96.0%, which was comparable to molecular diagnostics for culture-positive cases, but it could even produce reliable results for culture-negative cases.

**Fig. 3:**
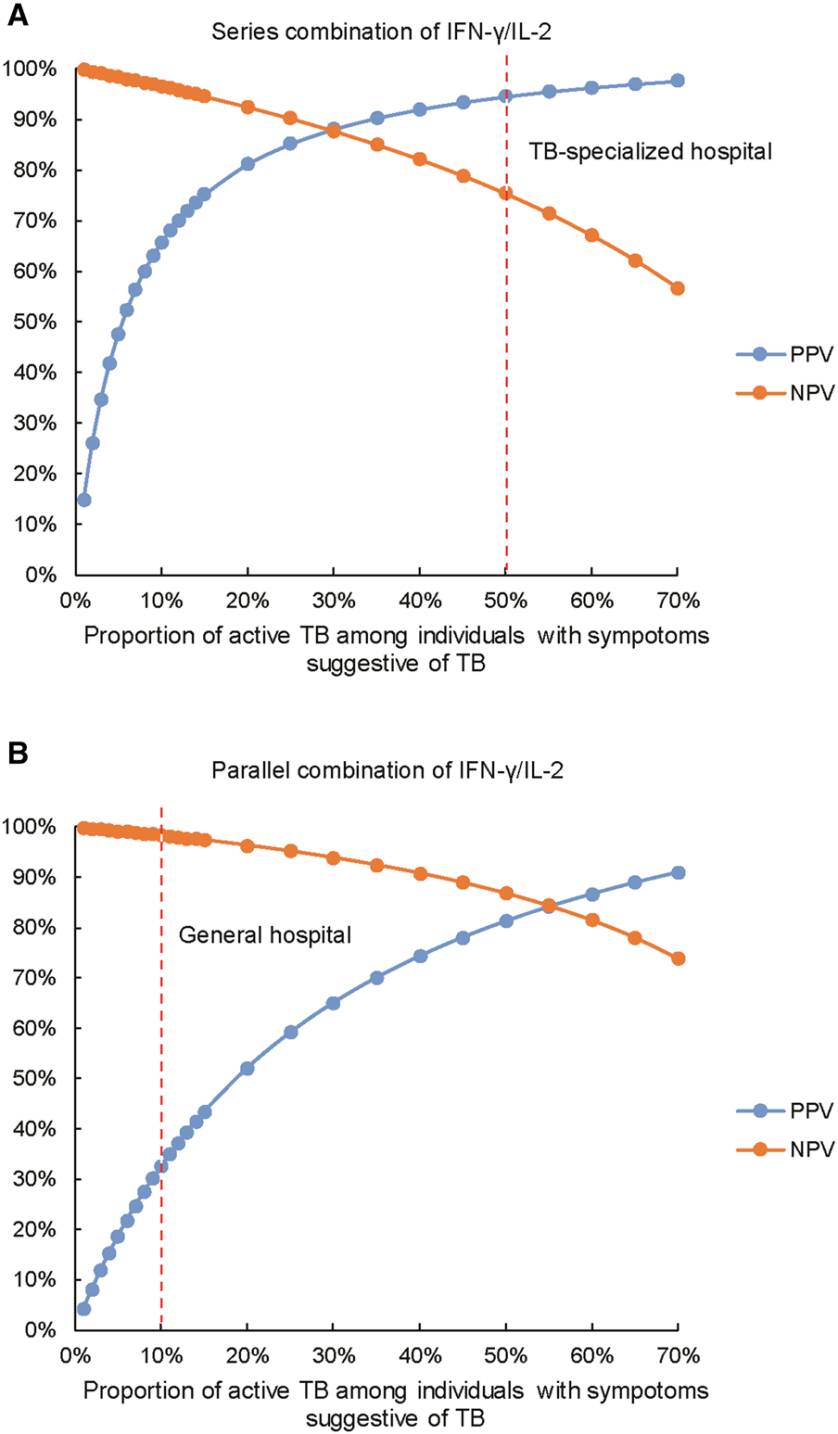
Positive predictive value and negative predictive value for TB detection using parallel and series combination of IFN-γ/IL-2 according to varying proportions of active TB among individuals with symptoms suggestive of TB

In view of the diagnostic utility of combined IFN-γ and IL-2 release assay, we modeled the false positive and false negative values for diagnosis of active TB with varying prevalence of active TB (Fig. 3), and proposed two diagnostic algorithms suitable for different hospital settings. For general hospitals in China which are in charge of screening tuberculosis suspects, approximate 10% of patients with symptoms suggestive of TB these TB suspects are finally diagnosed as having active TB [22]. The use of parallel combination with higher sensitivity could help clinicians identify more patients at high risk of active TB compared with the conventional smear microscopy. For TB-specialized hospitals, the proportion of active TB among TB suspects arrives at 50% [23]. Thus, the series combination is endorsed to facilitate earlier diagnosis of active TB patients, thereby preventing empiric treatment that might undermine clinical effect.

Another interesting finding of our study is the correlation the diagnostic accuracy and severity of disease in the population. The sensitivity of series IFN-γ/IL-2 combination in definite TB cases was significantly higher than that in clinically diagnosed cases. Chee and colleagues reported that quantitative T cells response is associated with mycobacterial burden and disease activity [24], whereas the conflicting results were observed in another experimental study that suggested lack of a clear correlation between antigen burden and T cell response [25]. Our results are consistent with the former that the enhanced stimulation with the increasing MTB-specific antigens results in stronger immunoregulatory cytokine production among active TB patients. Further research is needed to investigate the dynamic of MTB-specific IFN-γ/IL-2 secretion during anti-TB treatment.

There were several obvious limitations to the present study. First, despite using strict criteria, some patients affected with respiratory illness may be improperly diagnosed as active TB due to lack of laboratory support. The underlying classification error would have a negative impact on the reliability of our study conclusions. Second, there is strong evidence that the comorbidities, such as diabetes and immunosuppression status, are considered risk factors for negative immune response. However, the data of these patients were not collected, thus we could not evaluate their role on clinical performance of our immunological assay. Third, we evaluated only its diagnostic utility in pulmonary TB cases. Therefore, it is meaningful to confirm its performance among extrapulmonary TB cases in the future.

## Conclusions ant potential impact

In conclusion, we develop a new immunological method that can differentiate between active TB and other pulmonary diseases. Our data demonstrates that the various IFN-γ/IL-2 combinations provides promising alternatives for diagnosing active TB cases in different settings. More efforts should be paid to improve the operational convenience and cost effectiveness of this assay, which is essential for its scale-up in countries with a high TB burden.

## Data Availability

The datasets used and/or analysed during the current study available from the corresponding author on reasonable request.

## Abbreviations

CI: Confidence interval
ELISA: Enzyme-linked immunosorbent assay
IFN-γ: Interferon-γ
IL-2: Interleukin-2
IGRAs: Interferon-γ release assays
LTBI: Latent tuberculosis infection
MTB: *Mycobacterium tuberculosis*
OR: Odds ratio
PHA: Phytohemagglutinin
PBMCs: Peripheral blood mononuclear cells
ROC: Receiver operating characteristic
TB: Tuberculosis
Th-1: Type 1

## References

[1] Wang L, Zhang H, Ruan Y, Chin DP, Xia Y, Cheng S, Chen M, Zhao Y, Jiang S, Du X (2014). Tuberculosis prevalence in China, 1990–2010; a longitudinal analysis of national survey data. Lancet.

[2] World Health Organization (2020). Global tuberculosis report 2020.

[3] Walzl G, McNerney R, du Plessis N, Bates M, McHugh TD, Chegou NN, Zumla A (2018). Tuberculosis: advances and challenges in development of new diagnostics and biomarkers. Lancet Infect Dis.

[4] Whitworth HS, Badhan A, Boakye AA, Takwoingi Y, Rees-Roberts M, Partlett C, Lambie H, Innes J, Cooke G, Lipman M (2019). Clinical utility of existing and second-generation interferon-gamma release assays for diagnostic evaluation of tuberculosis: an observational cohort study. Lancet Infect Dis.

[5] World Health Organization (2014). Xpert MTB/RIF assay for the diagnosis of pulmonary and extrapulmonary TB in adults and children: WHO Policy update.

[6] World Health Organization (2016). The use of molecular line probe assay for the detection of resistance to isoniazid and rifampicin: policy update.

[7] Walzl G, Ronacher K, Hanekom W, Scriba TJ, Zumla A (2011). Immunological biomarkers of tuberculosis. Nat Rev Immunol.

[8] Diel R, Goletti D, Ferrara G, Bothamley G, Cirillo D, Kampmann B, Lange C, Losi M, Markova R, Migliori GB (2011). Interferon-gamma release assays for the diagnosis of latent *Mycobacterium tuberculosis* infection: a systematic review and meta-analysis. Eur Respir J.

[9] Sharma S, Kalia NP, Suden P, Chauhan PS, Kumar M, Ram AB, Khajuria A, Bani S, Khan IA (2014). Protective efficacy of piperine against *Mycobacterium tuberculosis*. Tuberculosis (Edinb).

[10] Menzies D, Pai M, Comstock G (2007). Meta-analysis: new tests for the diagnosis of latent tuberculosis infection: areas of uncertainty and recommendations for research. Ann Intern Med.

[11] Connell TG, Ritz N, Paxton GA, Buttery JP, Curtis N, Ranganathan SC (2008). A three-way comparison of tuberculin skin testing, QuantiFERON-TB gold and T-SPOT.TB in children. PLoS ONE.

[12] Armand M, Chhor V, de Lauzanne A, Guerin-El Khourouj V, Pedron B, Jeljeli M, Gressens P, Faye A, Sterkers G (2014). Cytokine responses to quantiferon peptides in pediatric tuberculosis: a pilot study. J Infect.

[13] Zhang L, Wan S, Ye S, Cheng X, Zhang Y, Shi X, Zhou B, Sun X, Liu X (2019). Application of IFN-gamma/IL-2 FluoroSpot assay for distinguishing active tuberculosis from non-active tuberculosis: a cohort study. Clin Chim Acta.

[14] Biselli R, Mariotti S, Sargentini V, Sauzullo I, Lastilla M, Mengoni F, Vanini V, Girardi E, Goletti D, Damelio R (2010). Detection of interleukin-2 in addition to interferon-gamma discriminates active tuberculosis patients, latently infected individuals, and controls. Clin Microbiol Infect.

[15] Xia H, Song YY, Zhao B, Kam KM, O'Brien RJ, Zhang ZY, Sohn H, Wang W, Zhao YL (2013). Multicentre evaluation of Ziehl-Neelsen and light-emitting diode fluorescence microscopy in China. Int J Tuberc Lung Dis.

[16] Tan Y, Su B, Cai X, Guan P, Liu X, Ma P, Zhou H, Liu J, Pang Y (2019). An automated smear microscopy system to diagnose tuberculosis in a high-burden setting. Clin Microbiol Infect.

[17] MacLean E, Broger T, Yerlikaya S, Fernandez-Carballo BL, Pai M, Denkinger CM (2019). A systematic review of biomarkers to detect active tuberculosis. Nat Microbiol.

[18] Frascella B, Richards AS, Sossen B (2020). Subclinical tuberculosis disease—a review and analysis of prevalence surveys to inform definitions, burden, associations and screening methodology. Clin Infect Dis.

[19] de Visser V, Sotgiu G, Lange C, Aabye MG, Bakker M, Bartalesi F, Brat K, Chee CB, Dheda K, Dominguez J (2015). False-negative interferon-gamma release assay results in active tuberculosis: a TBNET study. Eur Respir J.

[20] Millington KA, Innes JA, Hackforth S, Hinks TS, Deeks JJ, Dosanjh DP, Guyot-Revol V, Gunatheesan R, Klenerman P, Lalvani A (2007). Dynamic relationship between IFN-gamma and IL-2 profile of *Mycobacterium tuberculosis*-specific T cells and antigen load. J Immunol.

[21] Jeong YH, Jeon BY, Gu SH, Cho SN, Shin SJ, Chang J, Ha SJ (2014). Differentiation of antigen-specific T cells with limited functional capacity during *Mycobacterium tuberculosis* infection. Infect Immun.

[22] Dong G, Li X, Yang B, Zhang S, Li X, Tang Y, Zhang M, Yuan L (2016). SUI W: Analysis of the detectable rate of *Mycobacterium tuberculosis* in inpatients from respiration department of two comprehensive hospitals in Beijing. Chin J Antituberc.

[23] Liang Z, Song T, Liu Y, Li X, Liu G, Wu X, Zhou H, Zhang Y, Tan S, Liu Z (2017). Analysis of clinical diagnosis of 3315 cases with pulmonary tuberculosis suspicious symptoms transferred by non-tuberculosis control institutions. J Pract Med.

[24] Chee CB, KhinMar KW, Gan SH, Barkham TM, Pushparani M, Wang YT (2007). Latent tuberculosis infection treatment and T-cell responses to *Mycobacterium tuberculosis*-specific antigens. Am J Respir Crit Care Med.

[25] Pai M, Joshi R, Bandyopadhyay M, Narang P, Dogra S, Taksande B, Kalantri S (2007). Sensitivity of a whole-blood interferon-gamma assay among patients with pulmonary tuberculosis and variations in T-cell responses during anti-tuberculosis treatment. Infection.

